# Activation of astrocytes in the basal forebrain in mice facilitates isoflurane-induced loss of consciousness and prolongs recovery

**DOI:** 10.1186/s12871-023-02166-1

**Published:** 2023-06-20

**Authors:** Jialing Lin, Xuefeng Cheng, Haoyuan Wang, Lin Du, Xiangyu Li, Gaofeng Zhao, Chuangbo Xie

**Affiliations:** 1 Department of Anesthesiology, The Second Affiliated Hospital of Guangzhou University of Chinese Medicine, Guangzhou City, 510120 People’s Republic of China; 2 The Second Clinical College of Guangzhou University of Chinese Medicine, Guangzhou City, Guangdong Province, 510120 People’s Republic of China; 3 South China Research Center for Acupuncture and Moxibustion, Medical College of Acu-Moxi and Rehabilitation, Guangzhou University of Chinese Medicine, Guangzhou City, Guangdong Province, 510120 People’s Republic of China

**Keywords:** Basal forebrain, Astrocyte, DREADDs, Isoflurane anesthesia

## Abstract

**Objectives:**

General anesthesia results in a state of unconsciousness that is similar to sleep. In recent years, increasing evidence has reported that astrocytes play a crucial role in regulating sleep. However, whether astrocytes are involved in general anesthesia is unknown.

**Methods:**

In the present study, the designer receptors exclusively activated by designer drugs (DREADDs) approach was utilized to specifically activate astrocytes in the basal forebrain (BF) and observed its effect on isoflurane anesthesia. One the other side, L-α-aminoadipic acid was used to selectively inhibit astrocytes in the BF and investigated its influence on isoflurane-induced hypnotic effect. During the anesthesia experiment, cortical electroencephalography (EEG) signals were recorded as well.

**Results:**

The chemogenetic activation group had a significantly shorter isoflurane induction time, longer recovery time, and higher delta power of EEG during anesthesia maintenance and recovery periods than the control group. Inhibition of astrocytes in the BF delayed isoflurane-induced loss of consciousness, promoted recovery, decreased delta power and increased beta and gamma power during maintenance and recovery periods.

**Conclusions:**

The present study suggests that astrocytes in the BF region are involved in isoflurane anesthesia and may be a potential target for regulating the consciousness state of anesthesia.

## Introduction

General anesthesia, which is similar to sleep, is characterized by reversible loss of consciousness and is a powerful model for studying the state of consciousness [1]. In recent years, studies on the mechanism of general anesthesia have focused on neurons in certain brain regions and neural circuits [2]. However, there are few reports on the role of astrocytes (the largest number of glial cells in the brain) in general anesthesia [3]. Astrocytes support neurons, including neurotransmitter clearance, ion buffer, and energy supply, and play a critical role in “tripartite synapse” [4, 5]. Through astrocyte-neuron interaction, the activity of the surrounding neurons and synaptic transmission can be regulated by astrocytes [6–8]. Reportedly, astrocyte-derived adenosine triphosphate (ATP) can be degraded into adenosine and then act on surrounding neurons through the adenosine A1 receptor to inhibit neuronal activity, thereby promoting sleep [7, 9, 10].

Although astrocytes do not generate action potentials, they can communicate with neurons through the release of gliotransmitters triggered by intracellular “calcium waves” [11, 12]. The calcium activity of astrocytes is significantly different between anesthesia and arousal state, suggesting that the calcium activity of astrocytes is closely related to the state of anesthesia [13, 14]. As reported previously, the neuromodulatory system is involved in the change of the consciousness states [15]. Neuromodulators, such as acetylcholine and norepinephrine, can trigger the calcium activity of astrocytes and the subsequent downstream response, leading to the shift between resting and working activity states, which suggests that astrocytes participate in the regulation of consciousness state [15].

Moreover, studies have shown that anesthesia and sleep share common mechanisms [16]. The basal forebrain (BF) is a brain region that regulates the sleep-wake cycle and anesthesia-wake transition [17–19]. Cholinergic neurons and parvalbumin-positive GABAergic neurons in the BF can promote wakefulness, whereas somatostatin-positive GABAergic neurons facilitate sleep [17]. Inhibition of cholinergic neurons in the BF mediated loss of consciousness induced by anesthetics, whereas activation of cholinergic neurons promoted recovery from anesthesia [18]. In another study, the activation of somatostatin-positive neurons in the BF accelerated the induction of propofol and isoflurane and delayed recovery from anesthesia [19]. Intriguingly, an increasing number of studies have focused on the role of astrocytes in sleep regulation. Astrocytes can interact with surrounding neurons via ATP/adenosine to produce a sleep-promoting effect [7, 9, 10, 20]. This may occur similarly in general anesthesia, since administration of exogenous adenosine can promote the hypnotic effect of anesthetic drugs [21] and the A1 receptor agonist prolongs recovery time from anesthesia [22]. In addition, administration of caffeine, an adenosine receptor inhibitor, can accelerate arousal from anesthesia [23, 24]. However, whether astrocytes in the BF are involved in general anesthesia has not been illustrated yet. In the present study, we applied the designer receptors exclusively activated by designer drugs (DREADDs) approach [25, 26] to activate astrocytes in the BF region and observed its influence on the hypnotic effect of isoflurane. On the other side, we used an astrocyte-selective gliotoxin, L-α-aminoadipic acid (L-α-AAA), [27] to selectively inhibit astrocytes in the BF and explored its effect on general anesthesia. We found that chemogenetic activation of astrocytes can enhance anesthetic effect, which manifested as acceleration of the isoflurane induction and prolongation of recovery from anesthesia. On the contrary, inhibition of astrocytes attenuated the hypnotic effect of isoflurane with a longer time for induction and a faster recovery.

## Methods

### Animals and grouping

In the present study, Aldh1l1-creERT2 mice were obtained from Professor Yongjun Chen of Guangzhou University of Chinese Medicine. C57 BL/6J mice were purchased from Zhejiang Vital River Laboratory Animal Technology Co., Ltd. Male mice (age, 2–3 months; body weight, 25–30 g) were used in this study. All mice were housed in cages under specific-pathogen-free conditions with a 12-h day/night cycle (lights on from 7:00 AM to 7:00 PM), an ambient temperature of 22–24 °C, and ad libitum access to food and water. The experimental protocols and procedures were approved by the Ethics Committee for the Care and Use of Research Animals of Guangzhou University of Chinese Medicine (no. 20,210,707,008). All efforts were made to minimize the number of animals used in the study and the pain of the animals. The study was carried out in compliance with the ARRIVE guidelines.

In DREADDs experiment, forty-four Aldh1l1-creERT2 mice were randomly allocated into four groups: hM3Dq-Saline group, hM3Dq-CNO group, mCherry-Saline group, and mCherry-CNO group. In hM3Dq-Saline group and hM3Dq-CNO group, recombinant adeno-associated virus was injected into the BF region to express designed G-protein-coupled receptor - hM3Dq. In mCherry-Saline group and mCherry-CNO group, the virus that without hM3Dq component was injected as control. In hM3Dq-CNO group, clozapine N-oxide (CNO) was injected intraperitoneally to activate hM3Dq. In mCherry-CNO group, CNO was administrated to exclude the effects of CNO and its metabolite. hM3Dq-Saline group and mCherry-Saline group were administrated normal saline as control. At the end of the experiment, immunofluorescence staining was used to verify the virus transduction. Data from mice with poor virus expression or wrong location would be discarded. In the experiment of astrocyte inhibition, sixteen C57 BL/6J mice were randomly allocated into two groups: L-α-AAA group and aCSF group, receiving injection into BF region with L-α-aminoadipic acid (L-α-AAA) and artificial cerebrospinal fluid (aCSF), respectively.

### Stereotaxic microinjection and electroencephalogram(EEG) electrodes implantation

Mice were induced in an induction chamber with 1.5% isoflurane to anesthesia state and then transferred and stably fixed on a stereotaxic frame (RWD Life Science, Shenzhen, China), then kept anesthetized by 0.5 − 1.5% isoflurane through a mask to maintain stable a physiological state during the whole surgery process. Erythromycin ophthalmic ointment was used to protect the eyes. The scalp was locally anesthetized with 1% lidocaine before being sagittally cut. A heating mat was used to keep the mice warm. In the DREADDs experiment, recombinant adeno-associated viruses (rAAV-EF1α-DIO-hM3Dq-mCherry, AAV2/9 or rAAV-EF1α-DIO-mCherry, AAV2/9) (Brain-VTA, Wuhan, China) were then microinjected into the BF region (anteroposterior [AP], 0.25 mm; mediolateral [ML], ± 1.55 mm; dorsoventral [DV], −4.8 mm) of Aldh1l1-creERT2 mice using a microinjection pump (RWD Life Science) and a Hamilton microsyringe. The injection volume was 300 nL per side, and the injection rate was 30 nL/min. The microsyringe was maintained in place for 10 min before withdraw. Two to three weeks after virus injection, the mice were implanted with EEG electrodes. In the experiment of astrocyte inhibition, a solution of L-α-AAA (1 µL, 0.1 mol/L in aCSF) (A7275, Sigma, Germany) was injected into the BF region of C57 BL/6J mice to selectively inhibit astrocytes. In the control group, 1 µL of aCSF were injected using the same method. After the injection of L-α-AAA or aCSF, EEG electrodes were implanted.

Four stainless steel screws were screwed onto the surface of the dura as EEG electrodes: two recording electrodes were placed onto the frontal cortex (AP, 1.5 mm; ML, ± 1.5 mm), a ground electrode onto the left side of the parietal cortex (AP, −3 mm; ML, 2 mm), and a reference electrode onto the right side of the parietal cortex (AP, −3 mm; ML, −2 mm). Insulated silver wires were used to connect the screw electrodes and profile socket strips. All the components were fixed to the mouse head using dental cement. The stereotaxic coordinates of the mouse brain were determined using the mouse brain atlas by Paxinos and Franklin [28] during surgery.

### Drugs treatment

To induce the expression of hM3Dq and/or mCherry protein in astrocytes, Aldh1l1-creERT2 mice were injected intraperitoneally with tamoxifen (Sigma-Aldrich, Darmstadt, Germany) (20 mg/mL, 75 mg/kg body weight) from the second day after virus microinjection, once a day for 7 consecutive days. CNO (Brain-VTA, Wuhan, China) was dissolved in a 0.9% saline solution at a concentration of 0.3 mg/mL and was injected intraperitoneally (3 mg/kg body weight) one hour before the induction of anesthesia in the hM3Dq-CNO and the mCherry-CNO group. An equivalent volume of saline was administrated in the hM3Dq-Saline and the mCherry-Saline group. To explore the activation effect of DREADDs on astrocytes, CNO was administrated intraperitoneally 90 min before cardiac perfusion.

### Anesthesia experiment

The anesthesia experiments were performed between ZT6 and ZT12, 7 to 10 days after the EEG electrodes implantation. Before anesthesia experiment, all mice were handled and habituated to the experimental equipment (horizontal cylinder with a diameter of 12 cm and length of 20 cm) for 3 consecutive days, 30 min daily. The mice were anesthetized with 1.4% isoflurane in 100% O_2_ at 1.0 L/min, and the concentration of gases was monitored in real time. Experimenters rotated the cylinder 90° every 10 s until the mouse was in a four-limb-upward posture and could not turn itself prone, which was referred to as loss of the righting reflex (LORR). The time of recovery of the righting reflex (RORR), when the mouse returned to the prone position, was also recorded. Four time periods were defined for the entire procedure. The awake period was defined as the 20-min interval before initiation of isoflurane. The induction period was defined as the time from isoflurane onset to LORR. The anesthesia maintenance period was defined as the duration from LORR to RORR. The recovery period referred to the interval from the cessation of isoflurane administration to RORR.

### Analysis of EEG signals

EEG signals were recorded using an electrophysiological recording system (Medusa; Yige, Jiangsu, China) at a sampling frequency of 1000 Hz. EEG signals were collected 20 min before induction of isoflurane anesthesia and lasted continuously for 1 h in total. Only well-recorded EEG data were included for analysis. Raw data were bandpass-filtered at 0.5 to 60 Hz and further analyzed by using the Neuro Explorer version 4 software (Plexon, Dallas, TX, USA). The EEG signal was classified into five frequency bands: delta (0.5–4 Hz), theta (4–8 Hz), alpha (8–12 Hz), beta (12–25 Hz), and gamma (25–60 Hz). The spectrogram analysis function of the software was used to perform the power spectrum analysis. The relative power of each frequency band was analyzed for the four periods of isoflurane anesthesia.

### Immunohistochemistry and virus transduction verification

For glial fibrillary acidic protein (GFAP), Neun, and c-Fos staining, all mice were deeply anesthetized with isoflurane and cardially perfused with phosphate-buffered saline (PBS; pH, 7.4) and 4% paraformaldehyde in order. The brains were post-fixed in 4% paraformaldehyde overnight at 4 °C, then placed in 15% sucrose solution at 4 °C until they sank, and dehydrated in 30% sucrose solution. A cryostat microtome (Thermo Fisher Scientific, Waltham, MA, USA) was used to coronally slice the mouse brain into 40-µm sections. Sections containing the target brain area were washed with PBS thrice for 5 min each. Each brain slice was blocked with a 50-µL blocking solution (0.3% Triton X-100 and 5% bovine serum albumin) (Thermo Fisher Scientific) at 37 °C for 1 h 45 min. The primary antibodies, including rabbit anti-GFAP (PA5-85261, 1:500 dilution; Thermo Fisher Scientific), rabbit anti-NeuN (PA5-78639, 1:500 dilution; Thermo Fisher Scientific), and rabbit anti-c-Fos (2250s, 1:500 dilution; Cell Signaling Technology, Danvers, MA, USA), were diluted with blocking solution, added to brain slices, and incubated at 4 °C for 12 h. After incubation, the slices were washed with PBS thrice for 5 min each. The secondary antibodies, including donkey anti-rabbit Alexa Fluor 488 (A-21,206, 1:500 dilution; Thermo Fisher Scientific) was added to slices and incubated for 2 h at 37 °C. The slices were washed with PBS thrice and were mounted on glass slides. An anti-fade reagent with 4′,6-diamidino-2-phenylindole (DAPI) (Beyotime Biotechnology, Shanghai, China) was applied to seal the slices. Laser confocal microscopes (Nikon, Tokyo, Japan) were used to image all slices. All mice injected with viruses were verified by immunofluorescence staining for virus transduction, and data from those with incorrect injection location or poor virus transduction were excluded.

### Counting of hM3Dq^+^ and GFAP^+^ cells and c-Fos

The fluorescence labeling cells were counted on alternate sections on both sides of the brain in a 0.2 × 0.2-mm box in the BF region, including the horizontal limb of the diagonal band of Broca, lateral nucleus of the diagonal band of Broca, and substantia innominata [27]. Mice from each group were used to count the number of GFAP- (n = 5, 3–5 slices per mouse), mCherry- (n = 5, 3–5 slices per mouse), or c-Fos-positive (n = 3, 3 slices per mouse) cells, and DAPI labeling was used to identify the cells. Image J software (National Institutes of Health, Bethesda, MD, USA) was used to count the cells. We computed the penetrance of hM3Dq expression as (hM3Dq^+^ + GFAP^+^)/GFAP^+^ and the specificity of hM3Dq expression as (hM3Dq^+^ + GFAP^+^)/hM3Dq^+^.

### Statistical analysis

Statistical analysis was performed using the SPSS Statistics version 20 (IBM, NY, USA). In this study, the data are presented as the mean ± standard error of the mean. The Shapiro-Wilk test was used to test the normality of data distributions. The differences in the time of LORR or RORR between the chemogenetic activation and control groups were analyzed using two-way analysis of variance followed by the Bonferroni post hoc test. For the comparation of the time of LORR or RORR in the experiment of astrocyte inhibition, number of c-Fos positive cells, and EEG power ratio, we used unpaired t-tests or Mann-Whitney U test for data analysis when appropriate. *P* value < 0.05 was considered statistically significant in all cases.

## Results

### Astrocytes in the basal forebrain region can be specifically activated by the DREADDs approach

**Fig. 1:**
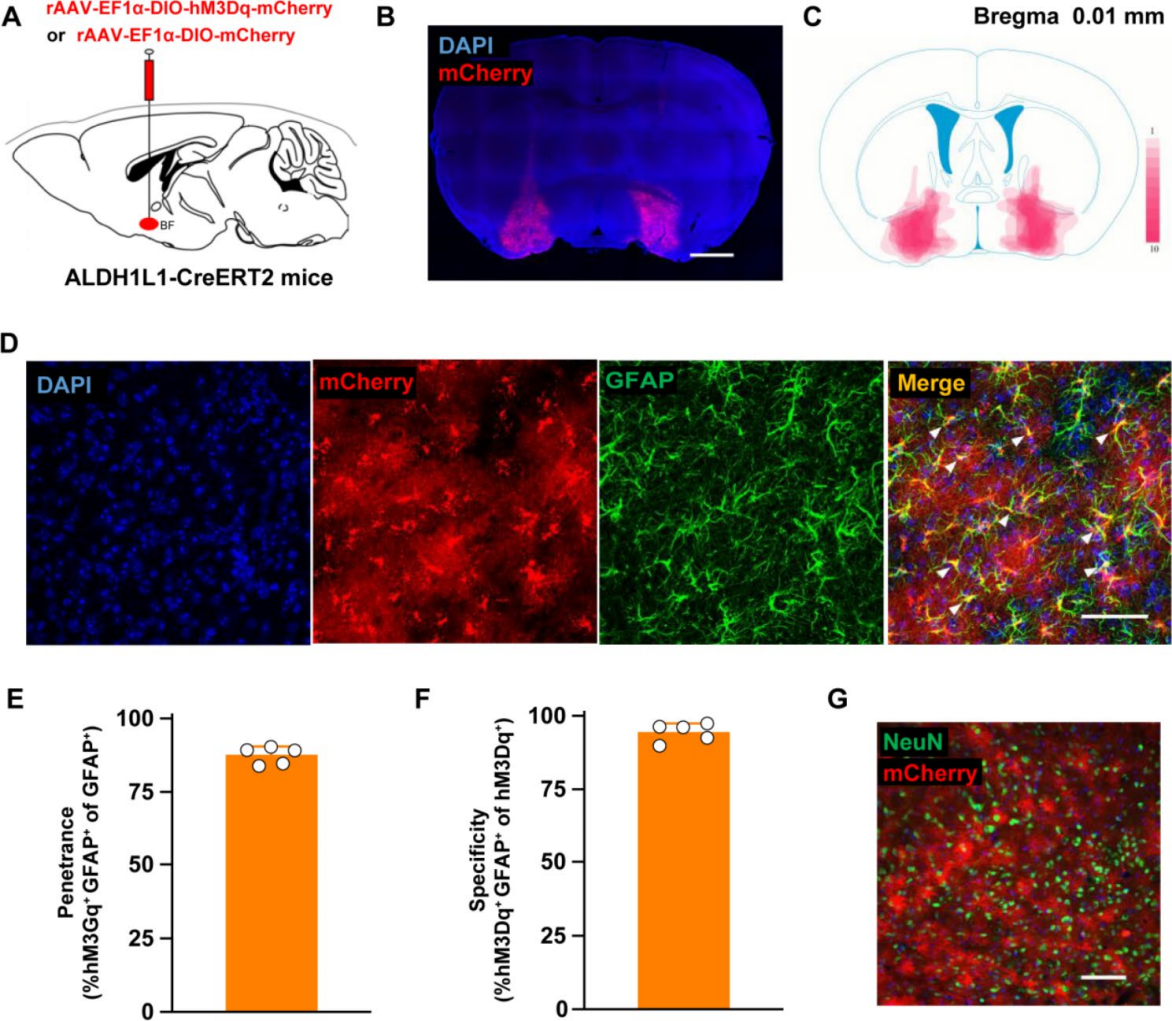
hM3Dq-mCherry is specifically expressed in astrocytes, but not in neurons. (**A**) Adeno-associated viral vectors with Cre-dependent expression of hM3Dq-mCherry or mCherry proteins (rAAV-EF1α-DIO-hM3Dq-mCherry or rAAV-EF1α-DIO-mCherry) are bilaterally injected into the basal forebrain of Aldh1l1-CreERT2 mice. (**B**) Representative image of viral expression in the basal forebrain of Aldh1l1-CreERT2 mice. (**C**) Overlay of hM3Dq-mCherry expression in the basal forebrain. Color depth represents the number of mice. (**D**) hM3Dq (red) is specifically expressed in astrocytes in the basal forebrain (scale bar, 50 μm). White arrows indicate the co-labeled of GFAP and mCherry. (E and F) hM3Dq is expressed in 87.54% of astrocytes (19 slices from 5 mice) (**E**), with the specificity of 94.62% (19 slices from 5 mice) (**F**). (**G**) Co-localization between mCherry and NeuN, the neuronal nuclear marker, is rarely detected (scale bar, 100 μm)

**Fig. 2:**
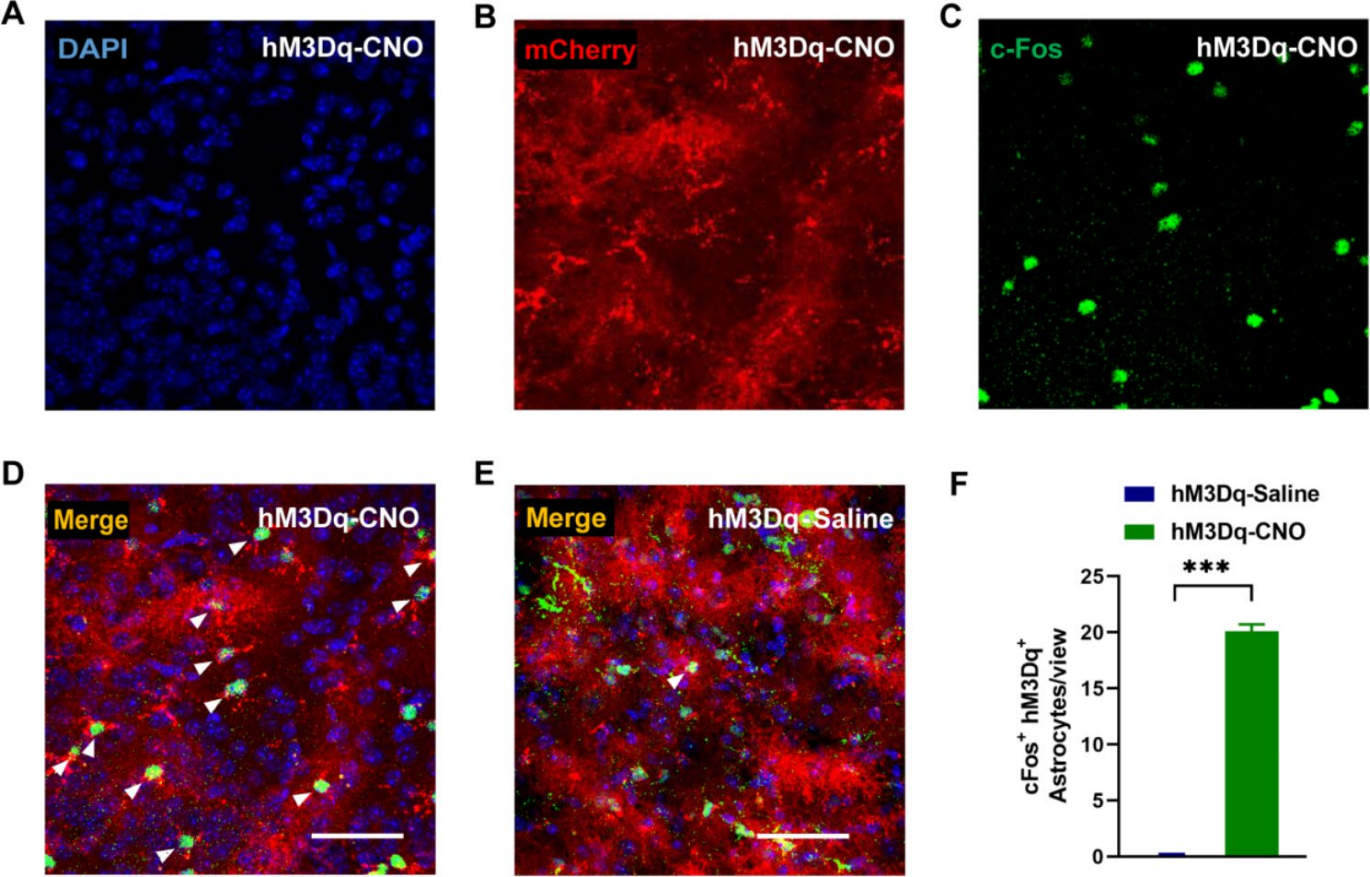
Clozapine N-oxide (CNO) specifically activates astrocytes that express hM3Dq. (**A-D**) Representative images of c-Fos expression (green) in the group with CNO administration, in which astrocytes are activated (scale bar, 100 μm). White arrows indicate the co-labeled of c-Fos and mCherry. (**E**) Representative images of c-Fos expression (green) in the group with saline administration, in which astrocytes are not activated (scale bar, 100 μm). White arrows indicate the co-labeled of c-Fos and mCherry. (**F**) CNO administration to mice expressing hM3Dq in basal forebrain astrocytes results in a significantly higher c-Fos expression than those administrated with saline. The asterisk indicates a significant difference (^***^P < 0.001; 9 slices from 3 mice)

To specifically activate astrocytes in the basal forebrain (BF) region, we injected a recombinant adeno-associated virus (rAAV-EF1α-DIO-hM3Dq-mCherry) into the BF of Aldh1l1-creERT2 mice (Fig. 1A). Virus-transduced proteins were expressed in the BF region (Fig. 1B C) and specifically merged with GFAP, an astrocyte marker (Fig. 1D). The infection rate (Fig. 1E) and specificity (Fig. 1F) of the virus were 87.54% and 94.62%, respectively. Notably, mCherry (reporter protein) did not merge with Neun, a neuronal biomarker (Fig. 1G). To activate astrocytes, we used CNO to exclusively activate the virus-transduced G-protein-coupled receptor, hM3Dq. After intraperitoneal injection with CNO, more astrocytes in the hM3Dq-CNO group expressed the immediate early gene protein, c-Fos, compared with the hM3Dq-Saline group (*P <* 0.05; Fig. 2A-2F).

### Activation of astrocytes in the BF region shortens the induction time of isoflurane anesthesia and prolongs the recovery time from anesthesia

**Fig. 3:**
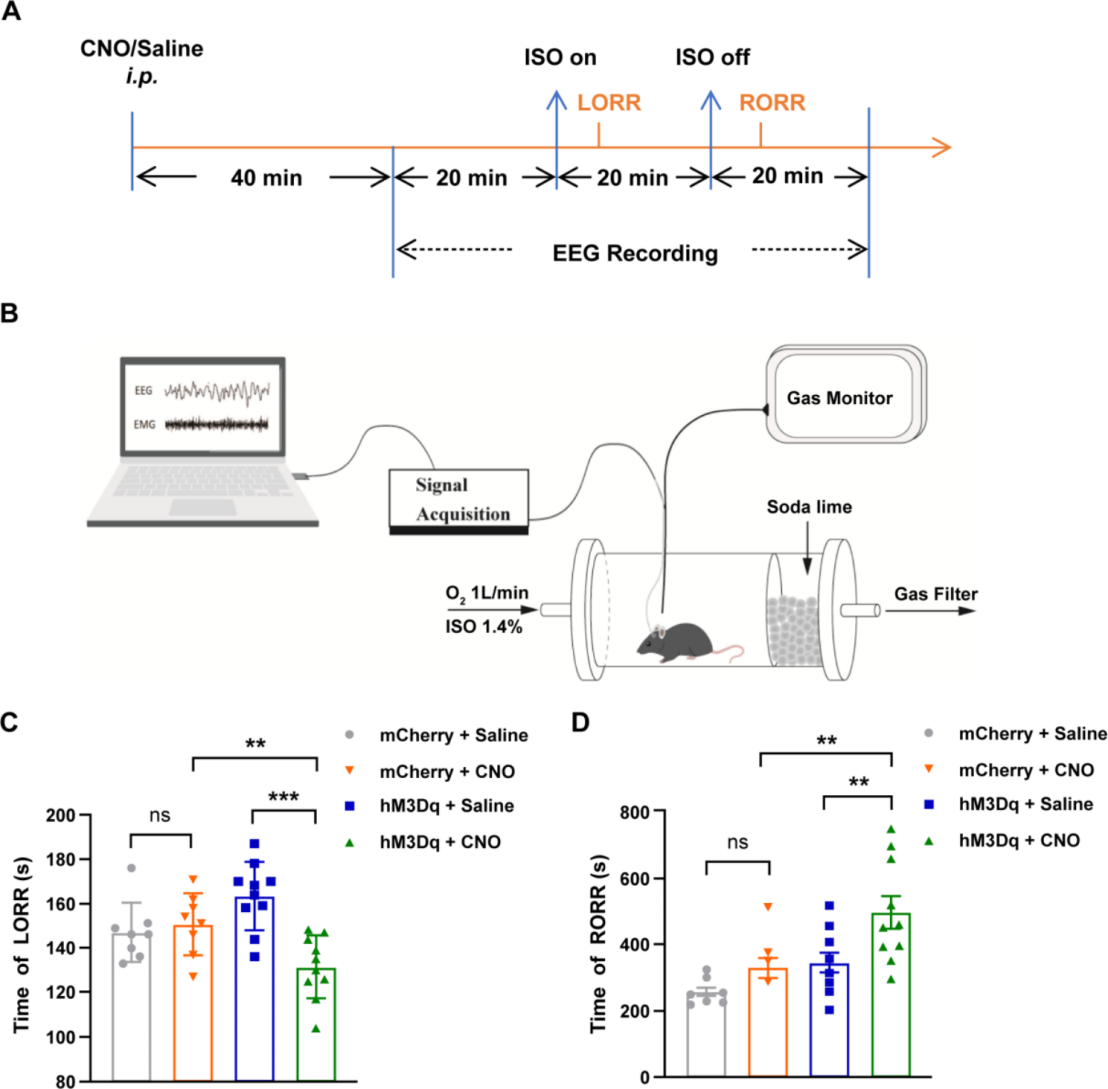
Activation of astrocytes in the basal forebrain region facilitates anesthesia-induced loss of consciousness and prolongs recovery. (**A**) Diagram of the loss of consciousness test and electroencephalogram recording in isoflurane anesthesia. (**B**) Schematic of experimental devices of isoflurane anesthesia. (**C**) Time of loss of righting reflex (LORR) with isoflurane exposure after intraperitoneal injections of saline or clozapine N-oxide (CNO) for 1 h (hM3Dq mice, n = 10; mCherry mice, n = 8). (**D**) Time of recovery of righting reflex (RORR) after isoflurane exposure ceased (hM3Dq mice, n = 10; mCherry mice, n = 8). Asterisks indicate significant differences (^**^P < 0.01, ^***^P < 0.001). ns indicates not being significant

To observe the effect of astrocyte activation in the BF on isoflurane anesthesia, the time of LORR and the time of RORR were recorded (Fig. 3A and B; Table 1). The hM3Dq-CNO group had significantly shorter LORR time than the hM3Dq-Saline group (131.50 ± 4.45 s vs. 163.40 ± 4.78 s; *P <* 0.001; Fig. 3C) and more prolonged RORR time (497.50 ± 48.84 s vs. 347.60 ± 29.51 s; *P <* 0.01; Fig. 3D). Meanwhile, the hM3Dq-CNO group had significantly shorter LORR time than the mCherry-CNO group (131.50 ± 4.45 s vs. 150.75 ± 4.96 s; *P <* 0.01; Fig. 3C) and longer RORR time (497.50 ± 48.84 s vs. 330.75 ± 29.50 s; *P <* 0.01; Fig. 3D).

To exclude the influence of CNO on the experimental results, we injected rAAV-DIO-mCherry-WPRE virus as a control virus and executed the same experimental process. We found that no significant difference in either the LORR time (150.75 ± 4.96 s vs. 147.13 ± 4.68 s; *P >* 0.05; Fig. 3C) or RORR time (330.75 ± 29.50 s vs. 258.38 ± 13.00 s; *P >* 0.05; Fig. 3D) between mice that received CNO and normal saline, respectively.

**Table 1:** Activation of astrocytes in the BF region facilitates anesthesia-induced loss of consciousness and prolongs recovery

Variables	mCherry + Saline group(n = 8)	mCherry + CNO group(n = 8)	hM3Dq + Saline group(n = 10)	hM3Dq + CNO group(n = 10)
**LORR** (s)	147.13 ± 4.68	150.75 ± 4.96^**^	163.40 ± 4.78^***^	131.50 ± 4.45
**RORR** (s)	258.38 ± 13.00	330.75 ± 29.50^**^	347.60 ± 29.51^**^	497.50 ± 48.84

Data are expressed as mean ± SEM. Compared with hM3Dq + CNO group, ^**^P < 0.01, ^***^P < 0.001

### Activation of astrocytes in the BF alters the power ratio of EEG spectrum

**Fig. 4:**
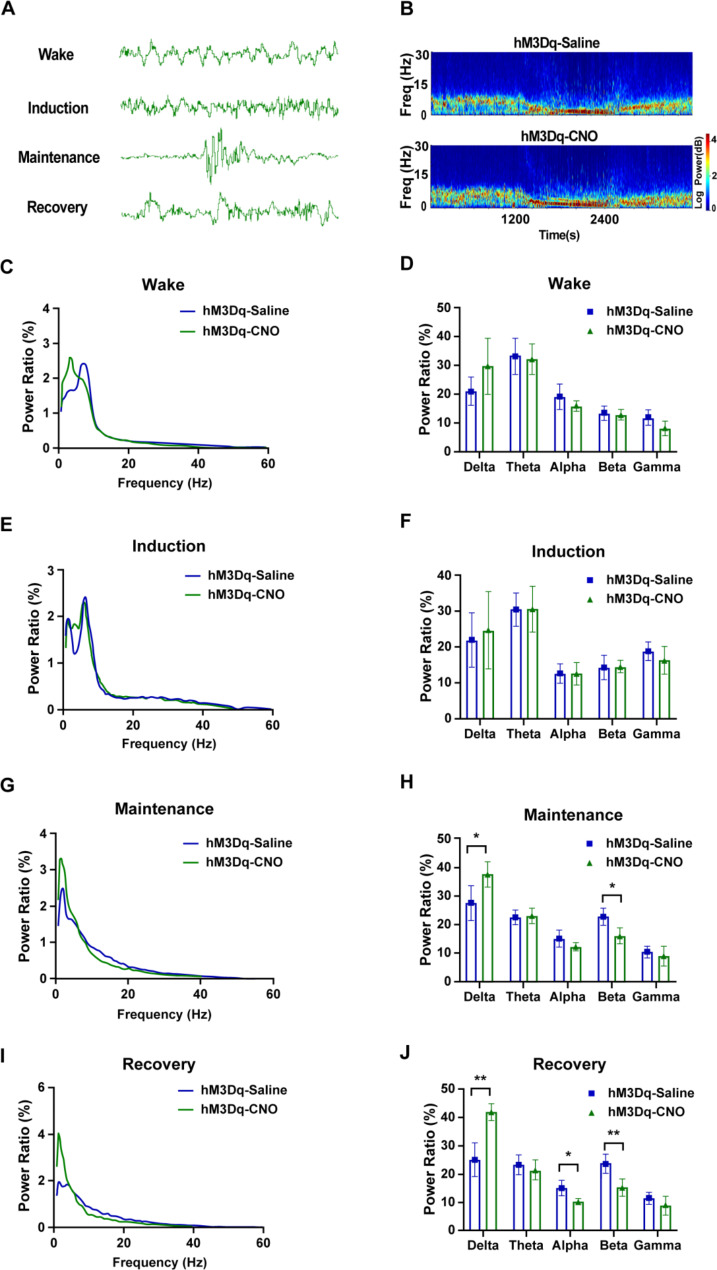
Activation of astrocytes in the basal forebrain region alters the power ratio of electroencephalogram (EEG) spectrum during anesthesia maintenance and recovery period. (**A**) Representative raw traces of EEG during different anesthesia periods. (**B**) Representative power spectrograms of EEG from two different groups. (**C, E, G, I**) Representative power ratio of EEG recording of isoflurane anesthesia during wake (**C**), induction (**E**), anesthesia maintenance (**G**), and recovery (**I**) periods. Blue line represents the hM3Dq mice injected with saline; green line represents the hM3Dq mice injected with clozapine N-oxide (CNO). (**D, F, H, J**) Comparison of EEG power ratios of different frequency bands (delta, theta, alpha, beta, and gamma) in different periods between the hM3Dq-Saline and hM3Dq-CNO groups (n = 4). The asterisk indicates a significant difference (^*^P < 0.05, ^**^P < 0.01).

To observe whether the activation of astrocytes in the BF region affects the power ratio of EEG frequency bands during isoflurane anesthesia, we recorded the EEG of mice during the experimental process (Fig. 3B). Various characteristics of EEG occurred in different anesthesia periods, burst suppression appeared during maintenance period (Fig. 4A). The power spectrum was analyzed (Fig. 4B-4J). The results showed that chemogenetic activation of astrocytes did not affect the power ratio of EEG in awake mice (*P >* 0.05; Fig. 4C D). During the induction period, there was also no significant difference in the EEG power spectrum between the two groups (*P >* 0.05; Fig. 4E F). The hM3Dq-CNO group had significantly higher delta power (*P <* 0.05; Fig. 4G H) and significantly lower beta power (*P <* 0.05; Fig. 4G H) during the anesthesia maintenance period than the hM3Dq-Saline group. The hM3Dq-CNO group had significantly increased delta power (*P <* 0.01; Fig. 4I J) and significantly decreased alpha and beta power (*P <* 0.05 and *P <* 0.01, respectively; Fig. 4I J) during the anesthesia recovery period, compared to the hM3Dq-Saline group.

### Inhibition of astrocytes in BF region delays anesthesia-induced loss of consciousness, promotes recovery, and changes the power ratio of EEG spectrum

**Fig. 5:**
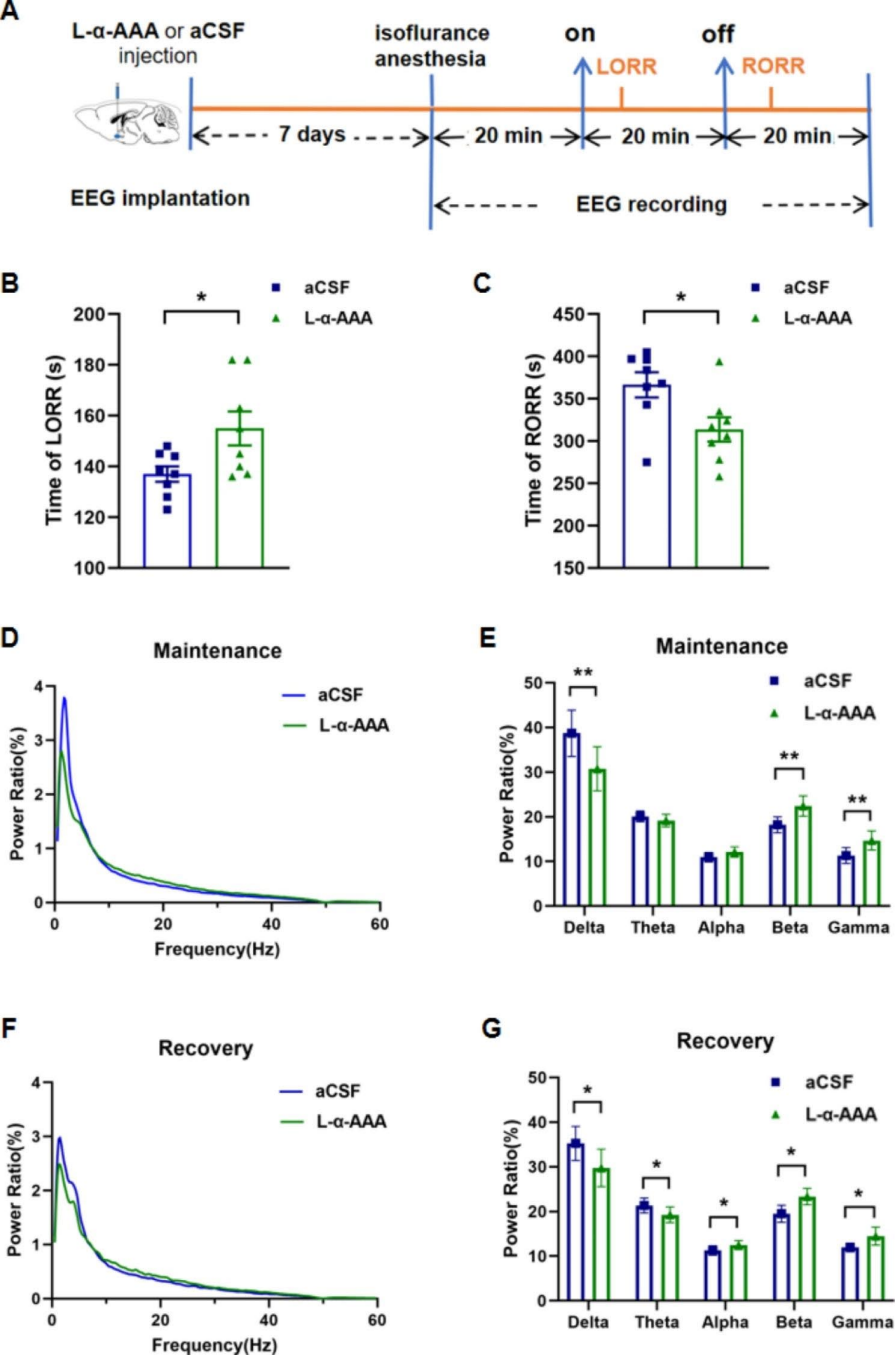
Inhibition of astrocytes in the BF region delays anesthesia-induced loss of consciousness and promotes recovery. (**A**) Diagram of astrocyte inhibitory experiment. (**B**) Time of loss of righting reflex (LORR) with isoflurane exposure (n = 8). (**C**) Time of recovery of righting reflex (RORR) after isoflurane exposure ceased (n = 8). (**D**, **F**) Representative power ratio of EEG recording of isoflurane anesthesia during anesthesia maintenance (**D**), and recovery (**F**) periods. Blue line represents aCSF group; green line represents L-a-AAA group. (**E**, **G**) Comparison of EEG power ratios of different frequency bands (delta, theta, alpha, beta, and gamma power bands) in different periods between the aCSF and L-a-AAA groups (aCSF group, n = 7; L-α-AAA group, n = 8). Asterisks indicate significant differences (^*^P < 0.05, ^**^P < 0.01)

To determine whether inhibition of astrocytes affects isoflurane anesthesia, L-α-aminoadipic acid (L-α-AAA), was injected into the BF region to selectively inhibit astrocytes, and the LORR and RORR times were recorded (Fig. 5A). The L-α-AAA group had significantly longer time of LORR (155.00 ± 19.02 s vs. 137.00 ± 8.69 s; *P <* 0.05; Fig. 5B) and shorter time of RORR (313.75 ± 40.89 s vs. 366.50 ± 42.30 s; *P <* 0.05; Fig. 5C) than that of the aCSF group (Table 2). Since activation of astrocytes in the BF altered the power ratio of EEG spectrum during the anesthesia maintenance and the recovery period, the changes induced by inhibition of astrocytes were analyzed as well. Compared with aCSF group, the EEG recordings of L-α-AAA group illustrated significantly decreased delta power (*P <* 0.01; Fig. 5D and E) and increased beta and gamma power (*P <* 0.01 and *P <* 0.01, respectively; Fig. 5D and E) during the anesthesia maintenance period. During the recovery period, L-α-AAA group showed significantly decreased delta and theta power (*P <* 0.05 and *P <* 0.05, respectively; Fig. 5F and G) and significantly increased alpha, beta and gamma power than that in aCSF group (*P <* 0.05, *P <* 0.05 and *P <* 0.05, respectively; Fig. 5F and G) .

**Table 2:** Inhibition of astrocytes in the basal forebrain region delays anesthesia-induced loss of consciousness and promotes recovery

Variables	aCSF group (n = 8)	L-α-AAA group (n = 8)
**LORR** (s)	137.00 ± 8.69	155.00 ± 19.02^*^
**RORR** (s)	366.50 ± 42.30	313.75 ± 40.89^*^

Data are expressed as mean ± SEM. Compared with aCSF group, ^*^P < 0.05

## Discussion

In the present study, we used the DREADDs approach to activate astrocytes in the BF and investigated its effect on isoflurane anesthesia. The results showed that activation of astrocytes in the BF could shorten anesthesia induction time (LORR time) and prolong recovery time from anesthesia (RORR time). The delta power of the EEG spectrum increased during the anesthetic maintenance and recovery periods, whereas beta power decreased significantly. On the other side, inhibition of astrocytes could delay isoflurane-induced loss of consciousness and promote recovery, with delta power decreased and beta and gamma power increased during maintenance and recovery periods.

Astrocytes, the most abundant glial cells in the brain, have been elucidated in several studies to possess far more functions than just supporting neurons. The finding of “tripartite synapse” [29, 30] and numerous studies [31–33] have confirmed that astrocytes and neurons are significantly closely interconnected to regulate brain functional activities. Halassa et al. [34] used fluorescent labeling to observe the three-dimensional structural relationship between astrocytes and neurons and found that a single astrocyte can wrap around neuronal somas and contact with 300 to 600 neuronal dendrites, indicating that astrocytes may become targets for regulating brain functional activities. Mediated by A1 receptors, astrocytes can regulate sleep homeostasis, suggesting that astrocytes participated in the regulation of consciousness states [9]. Kim et al. [7] reported that optogenetic activation of astrocytes in the ventrolateral preoptic nucleus, a sleep-regulating center, can promote sleep. Intriguingly, Pelluru et al. [20] found that optogenetic activation of astrocytes in the lateral hypothalamus, a brain region related to wake-promoting, can also promote sleep. The reversible loss of consciousness caused by anesthetics is similar to the sleep-wake state transition [10, 35]. In the present study, we investigated the role of astrocyte in the transition of states of consciousness.

To the best of our knowledge, this is the first time that the DREADDs approach (also called chemogenetics technique) was used to observe the effects of astrocyte activation on general anesthesia. The DREADDs approach can cause the activation of the downstream PLC/IP3 pathway, triggering the release of calcium from the endoplasmic reticulum and finally causing the release of gliotransmitters [24, 36, 37]. Chemogenetic activation of astrocytes has also been widely used to explore various brain functional activities [38, 39]. In the present study, we used the DREADDs approach to specifically activate astrocytes in the BF, and c-Fos expression confirmed that the astrocytes were activated. In addition, given that cholinergic, glutamatergic, and GABAergic neurons in the BF can affect the sleep-wake cycle and anesthetic-wake transition, to avoid the influence of neuronal activation on the experimental results, we combined the Aldh1l1-CreERT2 gene mouse [40] with adeno-associated viruses to specifically activate astrocytes. Our results showed that more astrocytes in the BF region could be activated in the chemogenetic activation group. Meanwhile, chemogenetic activation elements were rarely expressed in neurons, indicating that neurons are not chemogenetically activated.

Disruption of synaptic transmission is one of the mechanisms of loss of consciousness induced by anesthetics [41, 42]. Astrocytes had been demonstrated to inhibit synaptic transmission by releasing ATP/adenosine, which is mediated by A1 receptors [5, 43]. We speculated that chemogenetic activation of astrocytes in the BF may inhibit excitatory afferent neuron axons (such as orexinergic neurons from the lateral hypothalamus, noradrenergic neurons from the locus coeruleus, or dopaminergic neurons from the ventral tegmental area) to wakefulness-promoting neurons in the BF region [44]. Given that astrocytes can interact with hundreds of surrounding neurons, they may integrate information from different neurons and act on various types of neurons to exert a comprehensive effect in turn. More accurate mechanisms should be elucidated in future studies.

The inhibitory DREADDs technique [36]. (CNO activates hM4Di) is commonly used to inhibit the activity of neurons. However, it cannot exert an inhibitory effect on astrocytes because activation of hM4Di triggers the release of calcium from the endoplasmic reticulum, similar to activation of hM3Dq, but not inhibits [45, 46]. To determine the effect of inhibition of astrocytes on isoflurane anesthesia, we used a gliotoxin, L-α-aminoadipic acid, to selectively inhibit astrocytes in the BF. The results showed that inhibition of astrocytes can delay induction and promote recovery, which illustrated an opposite effect of astrocyte activation, further demonstrating the role of BF astrocytes in isoflurane anesthesia. We speculate that inhibition of astrocytes removed the inhibition of wake-promoting neurons in the BF and exerted a wake-promoting effect.

Isoflurane induced unconsciousness and sleep are two different states of unconsciousness that can be distinguished by their EEG profiles [47]. The EEG profile during the maintenance period of isoflurane anesthesia typically shows a burst-suppression pattern, characterized by periods of high-voltage activity (burst) followed by periods of low-voltage activity (suppression) (Fig. 4A). In contrast, sleep-induced unconsciousness is characterized by a more complex EEG profile. During non-REM sleep, the EEG shows slow-wave activity, which consists of high-amplitude, low-frequency delta oscillations (0.5-4 Hz). During REM sleep, the EEG shows a pattern of high-frequency, low-amplitude activity, which is similar to that seen during wakefulness. However, slow-delta oscillations are a shared feature of sleep and general anesthesia [47]. Sleep slow-delta oscillations are synchronous with relatively brief periods of inactivity in cortical neurons, whereas slow-delta oscillation in general anesthesia is induced or maintained with anesthetics that facilitate inhibitory post-synaptic currents (IPSCs). Similar to sleep, a mechanism to explain these GABAergic slow-delta oscillations likely involves loss of excitatory inputs from brainstem arousal centers to the cortex [47]. Slow-wave activity intensity was used to measure the depth of NREM sleep [46] and the total power percentages of frequency bands can be calculated to investigate the changes in depth of anesthesia [48]. Increased delta power represents a deeper anesthesia situation while beta and gamma power are related to wakefulness [18, 19, 48].

To explore whether astrocytes have an effect on the EEG spectrum, we monitored the EEG signals of the mice during anesthesia. We found that the activation of astrocytes in the BF had no effect on the EEG spectrum during the awake state, indicating that chemogenetic activation did not affect the basal EEG spectrum before anesthesia. However, delta power was increased and beta power was decreased during the anesthesia maintenance and recovery periods when the astrocytes in the BF area were activated, indicating that activation of astrocytes enhances the depth of general anesthesia. This result is in accordance with the elimination of cholinergic neurons in the BF area [18]. The main reason for such changes may be the decreased release of acetylcholine by BF cholinergic neurons in the cerebral cortex [18]. Notably, during the anesthesia recovery period, there is still a significant reduction in delta power, indicating that the effects of astrocyte activation on the state of consciousness are not transient; rather, they last longer, which may be related to the persistent absorption of CNO after intraperitoneal injection or due to the pattern of gliotransmitter release. Since activation of astrocytes in the BF had an effect on EEG spectrum during the anesthesia maintenance and the recovery period, we explored the EEG changes with inhibition of astrocytes during these two periods. Results showed that inhibition of astrocytes during anesthesia can lower delta power and rise beta and gamma power, facilitating the waking EEG and suppressing the lower frequency component. In cortex, activation of astrocytes can prolong sleep duration [47] and genetic ablation of astrocytic Ca^2+^ signal pathway impairs slow wave sleep [49]. However, given astrocytes have no long-projecting processes like the axons of neurons, we speculate that astrocytes in the BF influence the hypnotic effect of general anesthesia and EEG spectrum by regulating local neurons in the BF and exerting the effect on cortex EEG eventually.

In the present study, we proved that astrocytes in the BF are involved in regulating the hypnotic effect and EEG spectrum of isoflurane anesthesia. In future studies, we need to observe the effects of inhibition of gliotransmitter release and blockage of gliotransmitter receptors on isoflurane anesthesia. In addition, the dynamic changes in the calcium activity of astrocytes during the sleep-wake cycle are brain region-specific, [50] indicating that astrocytes may exert different effects in different brain regions. The specific role of astrocytes in other brain regions that related to general anesthesia needs to be further elucidated.

## Conclusion

Activation of astrocytes in the BF can facilitate isoflurane-induced loss of consciousness and prolong recovery. Inhibition of astrocytes can delay anesthesia induction and promote recovery. Astrocytes might play a crucial role in general anesthesia and may be a potential target for regulating consciousness states.

## Data Availability

The data used and analyzed in this study are available from the corresponding author on reasonable request.

## Abbreviations

ATP: adenosine triphosphate
BF: basal forebrain
CNO: clozapine N-oxide
DREADDs: designer receptors exclusively activated by designer drugs
EEG: electroencephalogram
GFAP: glial fibrillary acidic protein
GPCRs: G-protein-coupled receptors
LORR: loss of the righting reflex
RORR: recovery of the righting reflex
L-alpha-AAA: L-α-aminoadipic acid
